# Patient-derived tumor organoids: a new avenue for preclinical research and precision medicine in oncology

**DOI:** 10.1038/s12276-024-01272-5

**Published:** 2024-07-01

**Authors:** Lucie Thorel, Marion Perréard, Romane Florent, Jordane Divoux, Sophia Coffy, Audrey Vincent, Cédric Gaggioli, Géraldine Guasch, Xavier Gidrol, Louis-Bastien Weiswald, Laurent Poulain

**Affiliations:** 1 INSERM U1086 ANTICIPE (Interdisciplinary Research Unit for Cancers Prevention and Treatment), BioTICLA Laboratory (Precision Medicine for Ovarian Cancers), Université de Caen Normandie, Caen, France; 2 Comprehensive Cancer Center François Baclesse, UNICANCER, Caen, France; 3 Department of Head and Neck Surgery, Caen University Hospital, Caen, France; 4 ORGAPRED core facility, US PLATON, Université de Caen Normandie, Caen, France; 5 Biomics, CEA, Inserm, IRIG, UA13 BGE, Univ. Grenoble Alpes, Grenoble, France; 6 CNRS UMR9020, INSERM U1277, CANTHER Cancer Heterogeneity Plasticity and Resistance to Therapies, Univ. Lille, CNRS, Inserm, CHU Lille, Lille, France; 7 CNRS UMR7284, INSERM U1081, Institute for Research on Cancer and Aging, Nice (IRCAN), 3D-Hub-S Facility, CNRS University Côte d’Azur, Nice, France; 8 CNRS, INSERM, Institut Paoli-Calmettes, CRCM, Epithelial Stem Cells and Cancer Team, Aix-Marseille University, Marseille, France

**Keywords:** Cancer models, Tumour biomarkers, Cell biology

## Abstract

Over the past decade, the emergence of patient-derived tumor organoids (PDTOs) has broadened the repertoire of preclinical models and progressively revolutionized three-dimensional cell culture in oncology. PDTO can be grown from patient tumor samples with high efficiency and faithfully recapitulates the histological and molecular characteristics of the original tumor. Therefore, PDTOs can serve as invaluable tools in oncology research, and their translation to clinical practice is exciting for the future of precision medicine in oncology. In this review, we provide an overview of methods for establishing PDTOs and their various applications in cancer research, starting with basic research and ending with the identification of new targets and preclinical validation of new anticancer compounds and precision medicine. Finally, we highlight the challenges associated with the clinical implementation of PDTO, such as its representativeness, success rate, assay speed, and lack of a tumor microenvironment. Technological developments and autologous cocultures of PDTOs and stromal cells are currently ongoing to meet these challenges and optimally exploit the full potential of these models. The use of PDTOs as standard tools in clinical oncology could lead to a new era of precision oncology in the coming decade.

## Patient-Derived Tumor Organoids: Revolutionizing Oncology Research and Precision Medicine

The shift from 2D to 3D cell cultures has greatly improved cancer research, providing a more realistic model of tumors. Patient-Derived Tumor Organoids (PDTOs) have become a key tool in cancer research, allowing scientists to grow efficiently tumor cells from patient samples in a 3D environment that closely mirrors the original tumor. PDTOs are a major step forward in cancer research, bridging the gap between traditional cell cultures and clinical realities, with the potential for successful clinical applications despite some challenges that could be overcome by technological developments. Thus, they offer a promising platform for understanding cancer, testing drug responses, and developing personalized treatments, with the potential to greatly impact future patient care.

This summary was initially drafted using artificial intelligence, then revised and fact-checked by the author.

## Background

### Models in oncology: from 2D to 3D culture

**Fig. 1:**
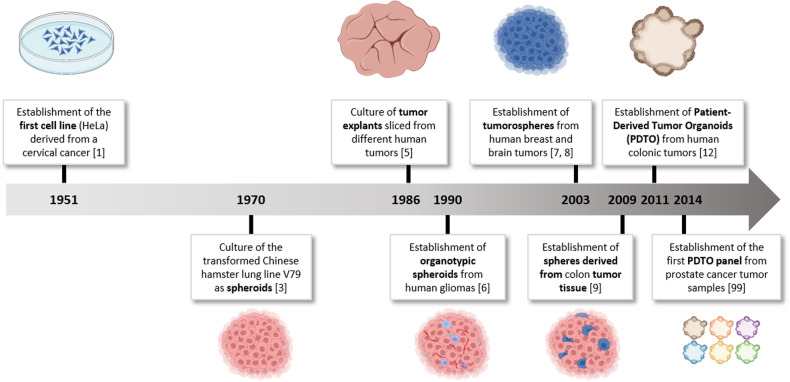
Timeline of the development of tumor cell models in oncology (created with BioRender.com). Adapted from^199^.

Since the establishment of the first cell line (HeLa) from a cervical cancer sample in 1951^1^, cell lines grown in monolayer cultures have served as tools to advance the understanding of cancer biology and to develop new treatments (Fig. 1). Although their ability to accurately mimic pathology is debated, they are still widely used in research laboratories. However, it is acknowledged that their genetic drift over time often prevents them from fully simulating real human tumors^2^. Their ability to mimic cellular interactions and the various gradients observed in vivo (such as oxygen, nutrients, and metabolites) are also compromised, ultimately affecting important cellular processes such as intracellular signaling pathway activation, adhesion, mechanotransduction, proliferation, and response to anticancer treatments, which does not consistently reflect the physiological reality of cancer tissue.

In this context, scientists have sought to maintain or recreate tumor complexity through various three-dimensional (3D) cell culture approaches. The spheroid model was proposed in the early 1970s by radiobiologists^3^. These highly compact spherical structures can reach a size of over 1 mm and are primarily obtained from immortalized cell lines, thus preventing tumor cell adhesion to the culture surface by using various methods (such as rotational culture systems and use of antiadhesive substrates, among other methods) to allow for cell aggregation^4^. Unfortunately, these cell lines acquire irrelevant mutations over time that do not reflect the biological characteristics of the original tissue.

Other 3D tumor cell culture approaches have subsequently emerged, including tumor explants obtained from slices of cancer tissue^5^, organotypic spheroids from patients’ tumor fragments cultured under nonadherent conditions^6^, tumorospheres generated from self-renewing tumor-initiating cells^7,8^, and tumor spheres from partially dissociated tumor tissue^9^. However, these models have limitations (such as limited culture maintenance, lack of proliferation, and low establishment success rates), thus explaining their disparate use in laboratories worldwide.

Over the past decade, the emergence of patient-derived tumor organoids (PDTOs) has progressively revolutionized 3D culture in oncology. Originally, culture conditions were optimized to allow for adult “normal” stem cells, which have self-renewal and differentiation properties, to self-organize in 3D and to reproduce the microanatomy and some functions of their original organ in vitro. The self-renewal capabilities of stem cells ensure the viability of the culture.

In 2009, the laboratory of Hans Clevers (Hubrecht Institute, Netherlands), a pioneer in this field, demonstrated that a single adult intestinal stem cell expressing the LGR5 receptor, which was isolated from mice, could reform in culture and exhibit a structure and cellular diversity that are similar to the crypts and villi of the intestinal epithelium^10^. These principles have since been adapted to many organs^11^ and to the culture of PDTO, initially based on digestive origin^12^ and subsequently from other cancerous locations^13^. Thus, PDTOs derived from various cancers, such as colorectal, lung, pancreatic, breast, ovarian, and prostate cancers, have been established by various teams (Table 1).

**Table 1:** Tumor types for which PDTOs have been established.

Tumor location		Histological type	Establishment rate (%)	Number of PDTO lines generated	Source of samples	References
Digestive	Appendix	High Grade and Low Grade appendiceal primary	73.1	19	S	^129^
		High Grade and Low Grade appendiceal primary	75	9	S	^165^
	Biliary Tract	IHC, gall bladder cancer, and NE carcinoma of the ampulla of Vater	33.3	6	S	^166^
		EHC, gall bladder cancer	85.7	6	S	^167^
	Colorectal	UN	90	22	S	^18^
		UN	60	46	S	^168^
		ADK, NE	100	55	S and biopsy	^32^
		ADK	77	55	S and biopsy	^43^
		UN	76	13	S (liver metastasis)	^169^
		ADK	68	19	Ascite (mestastasis)	^170^
	Gastric	Various	> 50	46	S	^70^
		Various	76.60	44	S, biopsy and ascite	^171^
		ADK	92	11	Ascite (metastasis)	^172^
	Gastroenteropancréatic	Neuroendocrine	64.1	25	S and biopsy	^173^
		Neuroendocrine	88.9	8	S	^174^
		Neuroendocrine	16	5	S	^175^
	Liver	HCC, CC	47	8	S	^19^
		HCC	26	10	Biopsy	^176^
	Pancreas	ADK	75	103	S and biopsy	^100^
		ADK	62	52	S and biopsy	^68^
		IPMN	81	13	S	^177^
Gynecologic	Breast	IDC and ILC	>80	95	S	^71^
		IDC and ILC	87.5	UN	S and biopsy	^178^
	Endometrium	Endometrioid Carcinoma	100	4	S	^179^
		Endometrioid, Clear Cell and Serous Carcinoma	40	16	S	^180^
	Ovary	Various	83	5	S	^179^
		Various	65	56	S and biopsy	^22^
Head and Neck	Head and Neck	SCC	65	26	S	^93^
		SCC	30.2	13	S	^181^
	Oropharyngeal and esophagus	SCC	71.4	15	Biopsy	^182^
		SCC	80	25	Biopsy	^183^
		ADK	31	10	S	^184^
	Salivary gland	Various	84	24	S	^185^
		Various	19	7	S and biopsy	^186^
	Thyroid	Papillary carcinoma	7	UN	S	^187^
		Papillary carcinoma	77.6	38	S	^188^
Urologic	Bladder	Urothelial carcinoma	70	12	Biopsy	^20^
		Urothelial carcinoma	82	9	S and ascites	^189^
	Kidney	Clear Cell Renal Cell Carcinoma	74	25	S	^190^
		Clear Cell, Papillary and Chromophobe Renal Carcinoma	76.7	33	S	^191^
	Prostate	Adenocarcinoma	15-20	6	Biopsy (metastasis)	^101^
		NE	16	4	Biopsy (metastasis)	^192^
Others	Brain	Glioblastoma	91.4	53	S	^38^
		Glioblastoma	31.25	10	S	^193^
	Lung	NSCLC	94	18	S	^194^
		NSCLC and Small Cell Carcinoma	55.5	20	S and biopsy	^102^
	Peritoneal	Mesothelioma	100	2	S	^195^
		Mesothelioma	85.7	7	S and biopsy	^196^
	Skin	Melanoma	90	9	S	^128^
		Melanoma	73	22	S	^197^
		Oral mucosal melanoma	64	30	S	^198^

*ADK* Adenocarcninoma, *CC* Cholangiocarcinoma, *EHC* Extrahepatic Cholangiocarcinoma, *HCC* Hepatocellular carcinoma, *IDC* Invasive Ductal Carninoma, *IHC* Intrahepatic Cholangiocarcinoma, *ILC* Invasive Lobular Carcinoma, *NE* Neuroendocrine, *NK* Not Known, *NSCLC* Non Small Cell Carnicoma, *SCC* Squamous Cell Carcinoma, *S* Surgical specimen

This review provides an overview of the various aspects of PDTO production, their use and relevance for research and/or care in oncology, and the associated challenges.

## Origin of the PDTO and methods of establishment

### Patient sample type

**Fig. 2:**
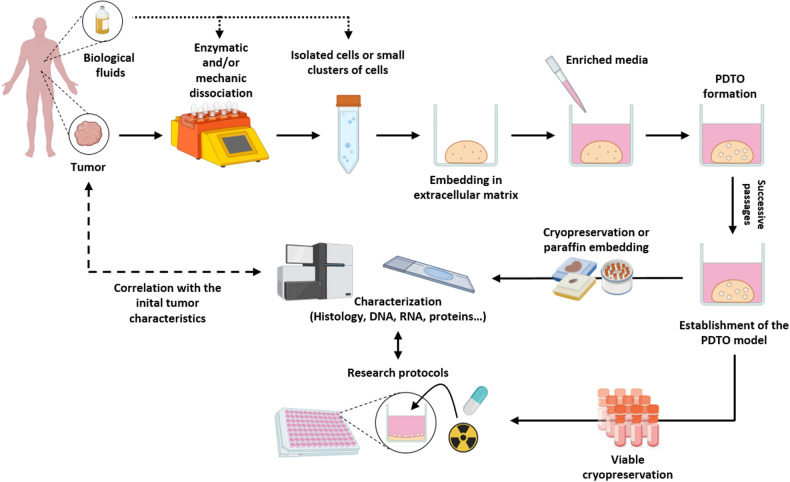
Procedure for the generation of PDTOs. Schematic representation of the various stages in the production of tumor organoids for research purposes (created with BioRender.com). Adapted from^199^.

PDTOs are generated by culturing tumor cells from patient biopsies, surgical specimens, or biological fluids such as ascites and blood^14,15^. In most cases, obtaining PDTOs from cancer tissues involves an initial step of mechanical and/or enzymatic dissociation, thus resulting in a suspension of isolated cells or small aggregates. The cells are then embedded in an extracellular matrix (ECM) dome and cultured in specific enriched media (Fig. 2) by using the submerged culture method^12^. PDTO can also be obtained by introducing tumorigenic alterations via genetic engineering^16^ in pluripotent stem cells, induced or embryonic stem cells, tissue-specific stem cells (adult stem cells), or normal organoids^17^.

### PDTO and tumor of origin

**Fig. 3:**
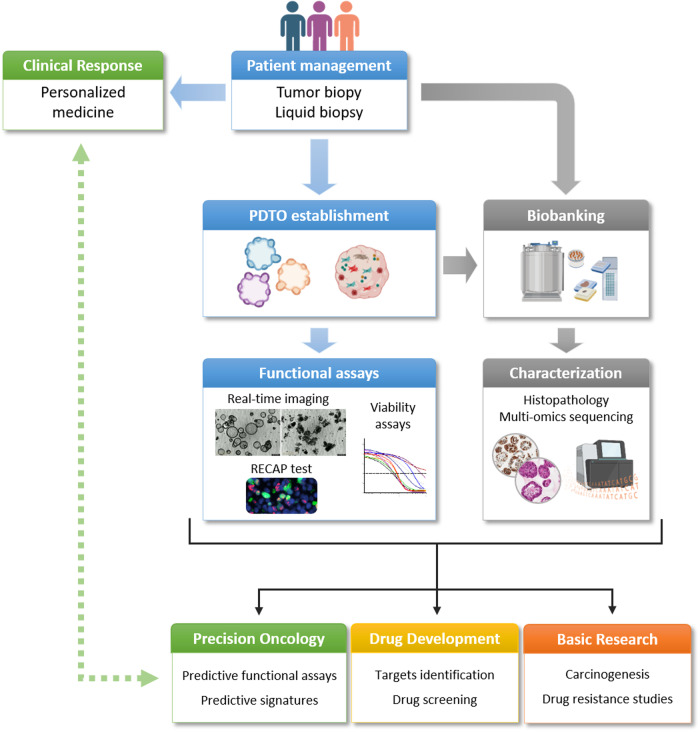
Contribution of PDTO to the fields of basic research and precision oncology (created with BioRender.com).

PDTOs have the advantage of being very similar to the tumor from which they are derived. For instance, PDTOs and tumor are comparable in terms of histology^18^ and genetics^19^ and display clonal evolution in culture^20^. However, principal component analysis (PCA) of transcriptomic data demonstrated that the parental tumors were grouped together and away from PDTOs from bladder cancer^20^, in contrast to PDX models, which are able to match with their tumor of origin^21^. These inconsistencies are mainly explained by the rapid growth of PDTOs in culture, as well as their lack of stromal components^20^. Overall, this resemblance remains relatively stable over time compared with that of cell lines^22^. Therefore, they are suitable for research and for predictive purposes in the context of precision medicine (Fig. 3). However, like any tumor sample harvested for diagnostic or predictive purposes, PDTOs represent only the tumor fraction from which they originate. Therefore, although the heterogeneity of the sample fragment is well preserved during the establishment of PDTOs (especially when they are truly generated from single cells representing the polyclonal nature of tumors in general^23^), other molecular characteristics that are present in another part of the tumor may be lost, thus emphasizing the importance of the quality of sampling during this process.

### Extracellular matrix

The ECM in which PDTOs are cultured provides an essential 3D microenvironment for their growth and self-organization. The most commonly used commercial ECMs are natural hydrogels derived from decellularized murine chondrosarcomas (Engelbreth-Holm-Swarm)^24^ with or without growth factors (Matrigel, BME). These hydrogels are primarily composed of laminin and collagen IV. However, these ECMs have many drawbacks, including significant interbatch variability that can affect repeatability and their animal origin, which may hinder their use in clinical settings. Additionally, their composition and their subsequent mechanical and chemical properties are not finely regulated, thus preventing the reproduction of topographical constraints specific to particular organs. Therefore, numerous natural and synthetic alternatives have been developed. Natural hydrogels include pure collagen hydrogels^25^ that may be mixed with other proteins, such as laminin, fibronectin, or hyaluronic acid^26^, as well as alginate hydrogels extracted from brown algae^27^. Protocols to obtain hydrogels from decellularized tissues that offer the biochemical properties of the original tissue have also been approved by the Food and Drug Administration (FDA) and have been proven to be effective for organoid culture^28^. Some laboratories have chosen to finely regulate the composition of their ECMs and have created synthetic hydrogels. The most commonly used polymers for these synthetic ECMs are polyethylene glycol (PEG)^29^ or poly(lactic-co-glycolic acid) (PLGA)^30^. Each of these hydrogels has advantages and disadvantages, and their use depends on the type of PDTO being cultured and the tissue of origin.

### Growth medium

The culture medium is supplemented with growth factors and signaling pathway inhibitors, the nature of which varies depending on the tissue’s origin to facilitate PDTO development^14^. Examples of routinely used media components are presented in Table 2. Two signaling pathways are essential for the growth of most types of PDTOs: activation of the EGFR pathway, which promotes cancer cell proliferation and requires supplementation with EGF in the culture medium, and stimulation of the Wnt pathway, which requires the addition of agonists (R-Spondin and Wnt3a) for LGR and Frizzled receptors, as well as their coreceptor LRP. This pathway is involved in controlling various processes, such as proliferation, adhesion, and cell differentiation, via stabilization of the β-catenin transcriptional co-factor^15,31^. However, although they are essential for the growth of colon organoids derived from healthy tissue^12^, in most colorectal cancer cells, the activation of mutations in the Wnt pathway eliminate the need to add Wnt and R-Spondin growth factors to the medium. Similarly, tumors with mutations in the EGF receptor signaling pathway are cultured in the absence of EGF^32–34^. Therefore, the choice of components for PDTO culture media depends on several established protocols, although additional experiments are needed to identify the optimal composition for each histological subtype of cancer.

**Table 2:** Main components of the media allowing for PDTO culture.

Tumor location		Reference	Medium Supplement	Antibiotics	Wnt pathway activator	Antioxydant	Precursor of NAD and NADP	EGFR activator	TGFß inhibitor	FGFR activator	CCK2 receptor agonist	BMPs inhibitor	ROCK Inhibitor	p38 MAPK inhibitor	Other
Digestive	Appendix	^129^	FBS	P/S	−	−	−	−	−	−	−	−	−	−	−
	Biliary Tract	^166^	B27, N2	−	RSPO	NAC	Nicotinamide	EGF	A83-01	−	Gastrin	−	Y-27632	−	Forsokolin
		^167^	B27, N2	P/S	Wnt3a, RSPO	−	−	EGF	A83-01	FGF10	Gastrin	Noggin	Y-27632	−	−
	Colorectal	^32^	B27	P/S	Wnt3a, RSPO	NAC	−	EGF	A83-01	–	Gastrin	Noggin	–	SB202190	–
		^18^	B27	Primocin	Wnt3a, RSPO	NAC	Nicotinamide	EGF	A83-01	–	Gastrin	Noggin	Y-27632	SB202190	PGE2
		^168^	B27	P/S	–	NAC	–	EGF	A83-01	–	Gastrin	–	Y-27632	SB202190	–
		^43^	B27, N2	ATB-ATM	Wnt3a, RSPO	NAC	Nicotinamide	EGF	A83-01	–	Gastrin	Noggin	–	SB202190	–
		^169^	–	P/S	Wnt3a, RSPO	–	–	EGF	-	FGF10, FGF2	–	Noggin	Y-27632	–	IGF-1, Endothelin 3
		^170^	B27	ATB-ATM, Gentamicin	–	–	–	EGF	A83-01	–	Gastrin	–	Y-27632	SB202190	SB431542
	Gastric	^70^	B27	P/S, primocin	Wnt3a, RSPO	NAC	–	EGF	A83-01	FGF10	Gastrin	Noggin	Y-27632	–	–
		^171^	B27	P/S	Wnt3a, RSPO	NAC	–	EGF	A83-01	FGF10	Gastrin	Noggin	–	–	–
		^172^	B27	P/S, primocin	Wnt3a, RSPO	NAC	Nicotinamide	EGF	A83-01	FGF10	Gastrin	Noggin	Y-27632	–	–
	Liver	^176^	B27, N2	–	Wnt3a, RSPO	NAC	Nicotinamide	EGF	A83-01	FGF10	Gastrin	–	–	–	HGF, Forsokolin
		^19^	B27, N2	P/S	Wnt3a, RSPO	NAC	Nicotinamide	EGF	A83-01	FGF10	Gastrin	Noggin	Y-27632	–	DXM, HGF, Forsokolin
	Pancreas	^68^	B27	P/S, primocin	Wnt3a, RSPO	NAC	Nicotinamide	EGF	A83-01	FGF10	Gastrin	Noggin	Y-27632	–	PGE2
		^100^	B27	–	Wnt3a, RSPO	NAC	Nicotinamide	EGF	A83-01	FGF10	Gastrin	Noggin	Y-27632	–	PGE2
		^177^	B27	P/S, primocin	Wnt3a, RSPO	NAC	Nicotinamide	EGF	A83-01	FGF10	Gastrin	Noggin	–	–	–
Gynecologic	Breast	^71^	B27	P/S, primocin	RSPO	NAC	Nicotinamide	–	A83-01	FGF10, FGF7	–	Noggin	Y-27632	SB202190	Neuregulin 1
	Endometrium	^179^	–	P/S, Amphotericin B	RSPO	–	–	EGF	–	–	–	Noggin	Y-27632	–	Jagged-1
	Ovary	^22^	B27	Primocin	Wnt3a, RSPO	NAC	Nicotinamide	EGF	A83-01	FGF10	–	Noggin	Y-27632	–	HCT, Neuregulin 1, Forsokolin
Head and Neck	Head and Neck	^93^	B27	P/S, primocin	RSPO	NAC	Nicotinamide	EGF	A83-01	FGF10, FGF2	–	Noggin	Y-27634	–	PGE2, CHIR, Forsokolin
	Oropharyngeal and esophagus	^182^	B27, N2	–	Wnt3a, RSPO	NAC	Nicotinamide	EGF	A83-01	–	Gastrin	Noggin	Y-27633	SB202190	–
	Salivary gland	^185^	B27	P/S	RSPO	NAC	–	EGF	A83-01	FGF10	–	Noggin	Y-27635	–	DXM, CHIR
		^186^	B27	Primocin	RSPO	–	–	EGF	A83-01	–	–	Noggin	Y-27636	–	CHIR, R1881
	Thyroid	^187^	B27	P/S	Wnt3a	–	Nicotinamide	EGF	A83-01	FGF2	–	Noggin	Y-27637	–	VEGF-121
Urologic	Bladder	^20^	FBS	Primocin	–	–	–	–	–	–	–	–	Y-27632	–	–
	Kidney	^190^	B27	P/S	RSPO	NAC	Nicotinamide	EGF	A83-01	FGF10, FGF2	–	Noggin		SB202190	HCR, PGE2, Epinephrine
	Prostate	^192^	B27	Primocin	RSPO	NAC	Nicotinamide	EGF	A83-01	FGF10, FGF2	–	Noggin	–	SB202190	PGE2, Testosterone
Others	Brain	^38^	B27, N2	P/S	–	–	–	–	–	–	–	–	–	–	2-mercaptoethanol, Insulin, NEAAs, Neurobasal
	Lung	^194^	B27	Primocin	RSPO	NAC	Nicotinamide	–	A83-01	FGF10, FGF7	–	Noggin	Y-27638	SB202190	–
		^102^	B27, N2	P/S	–	–	–	EGF	–	FGF2	–	–	Y-27639	–	–
	Skin	^128^	FBS	P/S	–	–	–	–	–	–	–	–	–	–	–

*P/S* Penicillin-Streptomycin, *ATB-ATM* Antibiotic-Antimycotic, *DXM* Dexamethasone, *Gmax* Glutamax, *HCT* Hydrocortisone, *L-Glu* L-Glutamine, *NAC* N-Acetyl-L-Cysteine.

### Alternate PDO cultures

PDTOs can also be cultivated by using an air-liquid interface (ALI) culture system, which has the advantages of growing epithelial cells and maintaining microenvironment components, including fibroblasts and immune cells^35^. In the ALI technique, the tissue is very finely sliced and subsequently coated with collagen before being deposited on a filter, after which media that is poor in growth factor is added. In this system, the microenvironment can be retained for one month^36^. In rarer cases, PDTOs can be cultured without the use of ECM, either to reverse the polarity (apical-out polarity) of cystic organoids grown in ECM^37^ or to establish patient-derived glioblastoma organoids^38^. In this instance, glioblastoma samples were cut into ~1 mm diameter pieces and cultured in ultralow attachment plates containing fully defined serum-free media. The plates were then placed on an orbital shaker to facilitate PDTO formation and increase nutrient and oxygen diffusion^38^.

### Expansion and use of PDTO

Once formed, PDTOs cultivated in submerged ECM can be dissociated and reseeded for amplification for experimental use. PDTOs can also be cryopreserved for subsequent reculturing. The biobanking of these models allows for the creation of large biological collections that are useful for numerous applications in both basic and clinical research^15^ (Fig. 2). The establishment of extensive panels of PDTOs is a valuable way of investigating cancer heterogeneity. Furthermore, these collections can be built together with models derived from the same tumor, such as patient-derived xenografts (PDXs), thus offering a broad range of complementary experimental possibilities^39,40^.

PDTOs can be subjected to various treatments (chemotherapy, radiotherapy, or targeted therapies), and their responses to treatments can be evaluated. Various viability tests, such as the CellTiter-Glo^41–43^, CellTiter Blue^44^, MTS^45^, and CCK-8 assays^46^, are widely used. Cellular imaging techniques (with or without probes), as well as histology and/or immunohistochemistry, are also utilized. They can be used to study the morphology of PTDOs (including size, texture, or organelle structure^47,48^), metabolism (for example, by using optical imaging^49^ or mass spectrometry^50^), proliferation (Ki67 expression proportion^38^) or cell death (by using the viability ratio^51^ or caspase probes^52^), as well as the expression of specific proteins constituting potential therapeutic targets (such as PD-L1 for immunotherapy^53^). Moreover, they can be performed at the endpoint and in real time without sample deterioration by using nontoxic probes. They allow for the assessment of the intensity and/or localization of these processes within PDTOs so that the latter may be classified according to their response to treatments.

## Applications in oncology

### Mechanistic and basic insights

Organoids and PDTOs are increasingly being used within the scientific community, particularly for basic oncology research. Organoids have demonstrated their utility in modeling the stages of carcinogenesis in various types of tumors, including colon cancer^33^, breast cancer^54^ and pancreatic cancer^55^. They have been transformed into tumor organoids via the inactivation of tumor suppressor genes (such as TP53, PTEN, or APC) or the activation of oncogenes (such as KRAS) by using CRISPR/Cas9 technology. Additionally, the inhibition of gene expression via RNA interference approaches in tumor organoids has highlighted the involvement of SIRT5 in pancreatic cancer^56^ and ARGLU1 in gastric cancers^57^. The evaluation of the very early stages of transition from healthy to tumor organoids can help researchers to better understand the molecular mechanisms of tumor initiation and thereby reveal new early diagnostic biomarkers for cancers for which early diagnosis is still a challenge, such as pancreatic cancer^58^. Tumor organoid models may also be relevant for mimicking the genomic evolution of tumors, as was recently demonstrated by Lee et al., who studied genetic alterations occurring during bladder cancer tumor organoid culture compared with tumors developing in vivo^20^.

The assessment of the mechanisms of resistance to treatments is a leading area of application for tumor organoids due to initial evidence showing their potential to recapitulate the clinical response of the original tumor. Resistance mechanisms to conventional and targeted therapies are dynamic and sequential. They involve reversible phenotypic changes, such as transient senescence mechanisms^59^, metabolic reprogramming^60^, epigenetic changes^61^, modification of the tumor microenvironment, epithelial–mesenchymal transition^62^ and/or irreversible mutational changes^63^. These phenomena are difficult to observe in patients or animal models, as multiple sampling steps during patient management are often difficult to achieve. Tumor organoids can be used to track the sequence of resistance acquisition and identify the involved mechanisms in a reproducible and more relevant manner than can be achieved via 3D spheroid culture^64^. Moreover, by using imaging techniques coupled with capture systems, tumor organoids exhibiting different responses can be analyzed separately, thus enabling the assessment of the effects of a treatment on cell heterogeneity (and vice versa). Several strategies have recently been adopted to analyze resistance mechanisms by using PDTOs. One of them involves the molecular comparison of PDTOs derived from patients treated with neoadjuvant chemotherapy to PDTOs from treatment-naive tumors to identify signaling pathways that could be targeted with specific therapies^65^. Another strategy is to grow tumor organoids from PDXs treated with chemotherapy in mice to evaluate several parameters that are impossible to assess in vivo, including the secretion of extracellular vesicles following treatment^66^. Recently, we developed a model of acquired resistance to FOLFIRINOX, which is a combination of three chemotherapies, from PDTO derived from pancreatic adenocarcinoma^67^. We measured a set of parameters (ROS production, double-strand DNA breaks, apoptosis, mutational profiles, and stemness) throughout the process. This scenario allowed for the identification of key steps of acquired resistance to combined drugs, thus highlighting the reversible nature of these mechanisms. Finally, we demonstrated that tumor organoids are an excellent model for residual disease, which is another aspect of treatment resistance^67^.

### Identification of efficient treatments and/or new therapeutic targets

Organoid biobanks exhibit promises for identifying new therapeutic strategies, guiding the use of molecules in development, and drug repurposing. Several groups have utilized panels of PDTOs originating from different tumor types to screen therapeutic molecules. The feasibility of medium-throughput pharmacological screening was demonstrated by exposing PDTOs derived from colorectal cancers to 83 molecules, thus highlighting the association between the efficacy of various molecules and relevant genetic alterations related to targeted pathways. Screening of molecular libraries in PDTO models has also identified MTAP as a new target in pancreatic cancer^68^ and SIRT1 as a new target in bladder cancer^69^. In another study, 9 gastric cancer PDTOs were exposed to 37 molecules that are used in clinical practice and under development, thus showing good responses to targeted therapies that are already indicated for other cancers (such as a stemness STAT-3 target inhibitor or a CDK4/CDK6 inhibitor)^70^. Sachs et al. also evaluated the relevance of 6 molecules (at 21 different concentrations) that act in vitro on the human epidermal growth factor receptor (HER) signaling pathway; moreover, the majority of HER2-overexpressing PDTOs were sensitive to these molecules, and those not expressing HER2 were resistant. However, some HER2-expressing lines did not meet this criterion, thus highlighting the value of functional tests to assess and predict treatment responses^71^. In another study, a panel of 24 pancreatic PDTOs showed variable sensitivity to 74 molecules, whether they were used in clinical practice or not; specifically, for the same PDTO model, responses to treatments targeting the same signaling pathways were similar^68^, thus demonstrating the consistency of the results. Another team used 6 PDTO models of rhabdoid tumors to identify, among 150 molecules, a potentially effective treatment for these rare pediatric tumors that currently have no therapeutic options. A molecule acting on neddylation (which is a posttranslational modification that adds the ubiquitin-like protein NEDD8 to substrate proteins) showed efficacy in all of the tested PDTO lines, thus indicating that NEDD8 is a promising target for further preclinical studies^72^. Ovarian PDTOs have also been used to validate the antitumor effect of a combination of a Bcl-x_L_ inhibitor with an EGFR inhibitor^73^ or with an α1-adrenergic receptor antagonist^74^. UBE2N has also been identified as being a potential therapeutic target in ovarian cancers, with its inhibition sensitizing several PDTO models to carboplatin^75^. Finally, coculture of PDTOs from glioblastoma with chimeric antigen receptor-T (CAR-T) cells demonstrated antigen recognition, subsequent T-cell activation, and tumor cell death, thus highlighting the potential of PDTOs for testing antigen-specific CAR-T-cell treatment responses^38^.

By recapitulating tumor heterogeneity and imitating the characteristics of the original tumor, the PDTO model allows for high-throughput screening of numerous emerging therapeutic options, thus making it potentially possible to identify tumor subtypes that could preferentially benefit patients. However, it is important to keep in mind that PDTO media often contain numerous growth factors and compounds, which can interfere with the evaluation of sensitivity to specific targeted therapies or anticancer drugs. For instance, the presence of EGF in the media may affect the use of EGFR-targeted drugs, such as cetuximab. Thus, the addition of exogenous EGF confers cetuximab resistance to colorectal cancer cell lines and PDTO^76^, and EGF-depleted media is needed to assess the response to this drug^77^. Therefore, screening a library of potential anticancer compounds may lead to a higher rate of false-positive results than expected. Moreover, high-throughput screening of PDTO models is a particularly burdensome, time-consuming, and costly process compared to cell line screening. To generalize its use, its benefits need to be clarified (which is ongoing in relevant laboratories), and efforts should be made to automate the culture, treatment, and analysis processes.

### Identification of biomarkers and predictive molecular signatures

PDTO panels can also be used to define predictive molecular signatures (genomic, transcriptomic, and proteomic signatures) of treatment response. In the context of conventional treatments, these approaches are performed directly on patients’ tumors. However, for molecules in development prior to clinical trials, it may be possible to define the sensitivity level of PDTOs to the molecules under investigation and to search for differential signatures in groups of sensitive or resistant PDTO models. This could lead to the very early development of companion tests that could support and accelerate the development of new drugs.

Several studies have established a link between response to conventional treatments and predictive signatures of various natures, thus providing perspectives for the development of innovative therapies or novel therapeutic sequences^78,79^. Biomarkers related to recurrence in pancreatic cancers have been identified by using a PDTO bank with established metabolic profiles. Several oncometabolites from the Krebs cycle were found to be more abundant in PDTOs from patients who experienced early recurrence. This characteristic may not only identify the most aggressive tumors but also constitute a vulnerability that could be targeted^80^. Machine learning analysis of pharmacogenomic data from collections of PDTOs derived from 19 colorectal cancer patients and 9 bladder cancer patients also identified specific biomarkers for sensitivity to 5-FU or cisplatin. These biomarkers subsequently demonstrated predictive value in discriminating responders and nonresponders in larger cohorts^81^. In-depth and exhaustive molecular characterization of extended collections of PDTOs derived from panels of tumors could efficiently enable the identification of predictive biomarkers (or predictive signatures including several of these biomarkers of different types) (Fig. 3). The value of this approach, which may include artificial intelligence techniques, will need to be confirmed by correlation studies with the clinical response of molecules in development. Nevertheless, the prospects in the field of developing new candidate drugs are immense, and such approaches could both accelerate their validation and provide a much better definition of patient subpopulations that are likely to benefit from these new therapies.

### Precision medicine

In addition to the availability of conventional or innovative treatments, precision medicine requires the identification of biomarkers enabling the selection of patients who are likely to benefit from these therapeutic strategies. Currently, the evaluation of the expression of key targets or the presence of genetic abnormalities associated with the responses to different treatments helps in guiding the therapeutic management of selected patients^82–84^. This information is of diagnostic, prognostic and predictive interest but also has several limitations, such as the lack of selectivity of some molecular signatures^85^ and the limits of interpretation, such as complex mutational signatures or variants of unknown importance^86^. This underscores the interest in developing functional tests that are capable of providing additional high-value information for predicting the response to both conventional and innovative treatments. The progressive implementation of functional tests in oncology began from the hypothesis that exposing primary cells from the patient’s tumor to treatments (isolated or not isolated from stromal cells) could predict their response. These tests could also identify correlations between ex vivo treatment responses and the presence of predictive biomarkers of different types (such as DNA, messenger RNA, noncoding RNA, and proteins, among other biomarkers) and origins (such as tumors, blood, and urine, among other origins). Thus, they may help in identifying the tumor phenotype through functional approaches that address various parameters of treatment response, and they can lead to the identification of predictive molecular signatures, which can correspondingly support the development of new therapies. The response of PTDO to one or more molecules after exposure can thereby be used predictively to guide therapeutic decisions for the patients that they originate from in a so-called “chemogram”, or they can even be retrospectively used (once a sufficiently large collection of models is obtained) to search for predictive molecular signatures (such as genomic, transcriptomic and proteomic signatures) of treatment response^87^.

A growing body of evidence indicates that PDTOs can predict the responses of the tumor that they derive from to anticancer treatments^88^. The correlation between the response to treatments of PDTO models and the clinical response of patients, which is a crucial and essential first step for the potential future clinical use of PDTOs, is becoming increasingly evident. A review reported of a sensitivity of 81% and a specificity of 74% for predicting treatment responses by using functional tests performed on PDTOs^89^. These figures are difficult to compare with other tests that are currently used in personalized medicine, such as the search for predictive mutations or aberrant expression profiles of tumor markers on which most targeted treatments are based. Indeed, the latter method requires a diagnostic test with a sensitivity and specificity as close as possible to 100% to be able to reach a “mutated” or “overexpressed” status. Once this status is determined, the treatment is administered to the patient, although not all selected patients will eventually respond. This scenario is precisely what is expected from functional tests that are performed on PDTO, which seek to directly determine the effectiveness of the treatment on the patient’s tumor without using an intermediate marker, which is an approach that includes (by definition) all or most of the parameters of the response to treatments.

Biomarkers can also be used to measure the response of PDTOs to treatment. For example, an increase in c-Jun phosphorylation after treatment exposure has been observed in cisplatin-sensitive gastric cancer PDTOs^90^. A pioneering study demonstrated the potential benefits of using PDTO derived from metastatic gastrointestinal tumors to predict the responses of 21 patients to different chemotherapies (100% sensitivity, 93% specificity, 88% positive predictive value, and 100% negative predictive value)^44^. According to another study, 91% of pancreatic cancer patients responded to first-line chemotherapy, and 80% of patients responded to second-line chemotherapy from PDTOs derived from 11 chemo-naive tumors. However, lines derived from 5 pretreated tumors predicted a treatment response in only 40% of patients^91^. Correlation with response to radiotherapy was analyzed in 19 colorectal cancer patients, thus resulting in the establishment of a prediction model with an accuracy of 82% for sensitive patients and 92% for resistant patients^92^. Furthermore, the least radiosensitive PDTO derived from head and neck squamous cell carcinomas in 7 patients corresponded to those who relapsed after treatment^93^. Finally, a study demonstrated an accuracy of 84% (78% sensitivity and 92% specificity) in predicting the response of colorectal PDTOs to a combination of chemotherapy and radiotherapy^51^; this was a particularly interesting result, which was due to the frequent use of multimodal treatments in clinical practice. Other studies have reported on the responses of ovarian cancer patients to PARP protein inhibitors, which are involved in the repair of single-strand DNA breaks. The use of these molecules is relevant in tumors with deficiencies in homologous recombination (HR) DNA repair, wherein the inhibition of single-strand break repair generates an accumulation of double-strand breaks, which remain unrepaired in this context. A functional assay known as the RECAP (REpair CAPacity test) provides an overall assessment of the status of the HR pathway. Before and after DNA damage induction by irradiation, the organization of repair foci through the HR pathway was quantified by detecting the localization of the RAD51 protein in proliferating cells. It has been applied to PDTO derived from ovarian tumors with potential success in identifying patients who are likely to benefit from treatment with PARP inhibitors^22,94^. Several clinical trials are underway to determine whether PDTO can predict patients’ responses to treatments (Table 3). Some research teams are continuing to use the response of PDTOs to guide therapeutic decision-making (Table 3). In one such study, PDTO was used to select the molecule administered after metastasectomy for recurrent colorectal cancer, thus leading to persistent remission at 6 months (when more than 50% of patients had progressed or died at that point in time)^95,96^. PDTO is also being used as a tool for therapeutic decision-making in patients with metastatic cancers who do not respond to first-line treatments for breast or gastrointestinal cancers (NCT04279509; NCT04450706; NCT04611035). They are also under evaluation for their predictive value in adjuvant settings for pancreatic cancer (NCT04931394) and breast cancer (NCT05177432), as well as in neoadjuvant settings for gastric (NCT05351398) or colorectal cancer (NCT04842006). Another ongoing study is even using PDTO to determine the drug to instill locally in bladder tumors during initial management (NCT05024734). Therefore, research offers hope for the rapid introduction of PDTOs into clinical management, which could even precede their use for drug screening in the pharmaceutical industry. However, their use for predictive purposes still has some limitations. For example, a study demonstrated a lack of correlation between the response to biopsy-derived PDTO and the clinical responses of patients with metastatic colorectal cancer treated with the 5-FU/oxaliplatin combination. Conversely, a prediction rate exceeding 80% was observed in patients treated with irinotecan and the irinotecan–5-FU combination, thus suggesting that the predictive nature of PDTO could depend on the anticancer agents that are used or that it would be necessary to better adapt the doses and ratios of chemotherapy combinations applied to the PDTO for them to remain relevant in an in vitro setting^97^. Furthermore, in another clinical study, patients with metastatic colorectal cancer that progressed despite first-line treatments were offered the opportunity to adapt their next therapeutic line based on the response of PDTOs that were derived from their tumor^98^. No improvements in clinical responses were observed; however, the amount of exploitable data was limited due to the low establishment rate (57%) and the overall status of the patients, who were too compromised to continue systemic treatment.

**Table 3:** Ongoing clinical trials comparing clinical and PDTO response to treatments or using PDTO to guide clinical decision-making.

Name of the study	Identifier	Estimated study completion date	Location	Type of cancer studied	Step of the treatment	Type of treatment
**Clinical trials in which principal outcome include comparison between PDTO and clinical response**						
Translational Analysis In Longitudinal Series of Ovarian Cancer Organoids (TAILOR)	NCT04555473	May-23	Roma, Italia	Ovary	NeoAdj. or Adj.	CT
Clinical Study on Drug Sensitivity Verification or Prediction of Therapy for Breast Cancer by Patient-Derived Organoid Model	NCT03544047	Jul-21	Beijing, China	Breast	NeoAdj.	CT
Drug Sensitivity Correlation Between Patient-Derived Organoid Model and Clinical Response in NSCLC Patients	NCT03453307	Jul-21	Shijazhuang, China	Lung	NeoAdj. or Adj.	CT, IT
Establishing Organoids From Metastatic Pancreatic Cancer Patients, the OPT-I Study	NCT03500068	Sep-22	Amsterdam, Netherlands	Pancreas	Pall.	CT
OPPOSITE: Outcome Prediction Of Systemic Treatment in Esophagogastric Carcinoma	NCT03429816	Aug-22	Dresden and Heidelberg, Germany	Gastric, œsophagus	NeoAdj.	CT, RT
Organoid Based Response Prediction in Esophageal Cancer (RARESTEM/Org)	NCT03283527	Jan-20	Groningen and Leewarden, Netherlands	Oeosphagus	NeoAdj.	CT, RT
Organoids in Predicting Chemoradiation Sensitivity on Rectal Cancer	NCT03577808	Nov-20	Shangai, China	CRC	NeoAdj.	CT, RT
Patient-derived Organoid Model and Circulating Tumor Cells for Treatment Response of Lung Cancer	NCT03655015	Dec-22	San Antonio, USA	Lung	Adj.	CT, IT
Pharmacotyping of Pancreatic Patient-derived Organoids	NCT05196334	Dec-24	Copenhague, Danemark	Pancreas	Prise en charge	CT
Cetuximab Sensitivity Correlation Between Patient-Derived Organoids and Clinical Response in Colon Cancer Patients.	NCT04906733	Dec-23	Shanghai, China	CRC	Adj.	CT, TT
Study on the Consistency Evaluation of Organoids Used in the Clinical Treatment of Ovarian Cancer With Anti-tumor Drugs	NCT05175326	Nov-21	Guangzhou, China	Ovary	NeoAdj. or Adj.	CT
Development of a Prediction Platform for Neoadjuvant Treatment and Prognosis in Pancreatic Cancer Using Organoid	NCT04777604	Jan-26	Seoul, Korea	Pancreas	NeoAdj.	CT
Organoids-on-a-chip for Colorectal Cancer and in Vitro Screening of Chemotherapeutic Drugs	NCT04996355	May-24	Beijing, China	CRC	Adj.	CT
Development of a Prediction Platform for Adjuvant Treatment and Prognosis in Resected Pancreatic Cancer Using Organoid	NCT04736043	Jan-26	Seoul, Korea	Pancreas	Adj.	CT
Study on Consistency Evaluation for Drug Sensitivity of Patient-Derived Organoid Model From Cholangiocarcinoma Patients	NCT05634694	Dec-24	Guangzhou, China	Cholangiocarcinoma	Adj.	CT
SOTO: Treatment Sensitivity of Organoids to Predict Treatment Outcome	NCT05400239	May-23	London, United Kingdom	HNSCC	Adj. or Pall.	CT, RT
The Culture of Advanced or Recurrent Ovarian Cancer Organoids and Drug Screening	NCT05290961	Dec-24	Chongqing, China	Ovary	Adj.	CT
The Culture of Ovarian Cancer Organoids and Drug Screening	NCT04768270	Dec-24	Chongqing, China	Ovary	Adj.	CT
Tailoring Treatment in Colorectal Cancer (TargetCRC)	NCT05401318	Jan-27	Viken, Norway	CRC	UN	CT, IT
3D Bioprinted Models for Predicting Chemotherapy Response in Colorectal Cancer With/Without Liver Metastases	NCT04755907	Dec-23	Beijing, China	CRC	NeoAdj. or Adj.	CT
KM3D Multicenter Cancer Consortium: Predicting Patient Response Using 3D Cell Culture Models	NCT05338073	Jan-26	Durham, USA	Various	UN	CT
**Clinical trials in which secondary outcome include comparison between PDTO and clinical response**						
Early-Line Anti-EGFR Therapy to Facilitate Retreatment for Select Patients With mCRC	NCT04587128	Oct-25	Madison, USA	CRC	Pall.	CT, TT
Establishment of Squamous Cell Organoids of the Head and Neck to Assess Their Response to Innovative Therapies (ORGAVADS)	NCT04261192	Feb-25	Caen, France	HNSCC	Adj.	CT, RT, IT
Feasibility Study of Multi-Platform Profiling of Resected Biliary Tract Cancer	NCT04561453	Jun-25	Chicago, USA	Biliary Tract	Adj.	CT
Novel 3D Myeloma Organoid to Study Disease Biology and Chemosensitivity (Organoid)	NCT03890614	Apr-23	Winston-Salem, USA	Myeloma	UN	CT
Patient-derived Organoids of Lung Cancer to Test Drug Response	NCT03979170	Dec-24	Geneva, Switzerland	Lung	Adj.	CT
Prediction Model of Response for CCRT in Esophageal Cancer	NCT03081988	Dec-22	Daegu, Korea	Esophagus	NeoAdj.	CT, RT
Real Time Molecular Analysis of Breast Cancer Receiving Neo-adjuvant Chemotherapy (NEO-R)	NCT04504747	Jan-30	Marseille, France	Breast	NeoAdj.	CT
Trifluridine/Tipiracil and Irinotecan for the Treatment of Advanced Refractory Biliary Tract Cancer	NCT04072445	Jan-23	Minessota, USA	Biliary Tract	Pall.	CT
Developing Breast (Cancer) Organoids	NCT05317221	May-28	Maastricht, Netherlands	Breast	UN	CT
Novel 3D Hematological Malignancy Organoid to Study Disease Biology and Chemosensitivity (Organoid)	NCT03890614	Apr-23	Winston-Salem, USA	Hematology	UN	CT
Consistency Between Treatment Responses in PDO Models and Clinical Outcomes in Gastric Cancer	NCT05203549	Jun-23	Shangai, China	Myeloma	NeoAdj., Adj., Pall.	CT
The Culture of Advanced/Recurrent/Metastatic Colorectal Cancer Organoids and Drug Screening	NCT05304741	Dec-23	Chongqing, China	CRC	Pall.	CT, TT
Establishment of an ex Vivo Tumor Collection of Triple-negative Breast Cancers in Order to Validate the Interest of Innovative Therapies and the Search for Predictive Biomarkers of Response to Treatment (TRIPLEX)	NCT05404321	Dec-26	Caen, France	Breast	NeoAdj.	CT
A Pilot Study of a Micro-Organosphere Drug Screen Platform to Lead Care in Advanced Breast Cancer	NCT04655573	Oct-24	Durham, USA	Breast	NaoAdj. or Pall.	CT
Using Ex Vivo Tumoroids To Predict Immunotherapy Response In NSCLC (TUMORIN)	NCT05332925	Feb-24	Kansas, USA	Lung	Pall.	IT
Molecular Characteristics of Gastroesophageal Adenocarcinoma (MOCHA): A Prospective Feasibility Study	NCT04219137	Nov-21	Toronto, Canada	Oesophagogastric	UN	UN
Systemic Neoadjuvant and Adjuvant Control by Precision Medicine in Rectal Cancer (SYNCOPE)	NCT04842006	Dec-31	Helsinki, Finlande	CRC	NeoAdj.	CT, RT
Clinical trials using PDTO to guide clinical decisions						
Functional Precision Oncology for Metastatic Breast Cancer (FORESEE)	NCT04450706	Aug-25	Salt Lake City, USA	Breast	Pall. (2nd line)	CT
Q-GAIN (Using Qpop to Predict Treatment for GAstroIntestinal caNcer)	NCT04611035	Jan-23	Singapore	Gastrointestinal	Pall. (2nd line)	CT (14 drugs)
Selecting Chemotherapy With High-throughput Drug Screen Assay Using Patient Derived Organoids in Patients With Refractory Solid Tumors (SCORE)	NCT04279509	May-22	Singapore	HNSCC, CRC, ovary, breast	Pall. (3rd line)	CT (10+/-5 drugs)
Organoid-Guided Chemotherapy for Advanced Pancreatic Cancer	NCT04931381	May-25	Shanghai, China	Pancreas	Pall. (1st line)	CT (5 drugs)
Organoid-Guided Adjuvant Chemotherapy for Pancreatic Cancer	NCT04931394	May-25	Shanghai, China	Pancreas	Adj.	CT (5 drugs), RT
Guiding Instillation in Non Muscle-invasive Bladder Cancer Based on Drug Screens in Patient Derived Organoids	NCT05024734	Nov-26	Berne, Suisse	Bladder	Adj.	CT intravesical (4 drugs)
Patient-derived-organoid (PDO) Guided Versus Conventional Therapy for Advanced Inoperable Abdominal Tumors	NCT05378048	Jul-25	Hong Kong	Abdominal Solid Tumor	Pall. (2nd line)	CT
The Clinical Efficacy of Drug Sensitive Neoadjuvant Chemotherapy Based on Organoid Versus Traditional Neoadjuvant Chemotherapy in Advanced Gastric Cancer	NCT05351398	Dec-23	Shanghai, China	Gastric	NeoAdj.	CT
The Clinical Efficacy of Drug Sensitive Neoadjuvant Chemotherapy Based on Organoid Versus Traditional Neoadjuvant Chemotherapy in Advanced Rectal Cancer	NCT05352165	Dec-25	Shanghai, China	CRC	NeoAdj.	CT (5 protocols)
Quadratic Phenotypic Optimization Platform (QPOP) Utilization to Enhance Selection of Patient Therapy Through Patient Derived Organoids in Breast Cancer (QUEST)	NCT05177432	Dec-25	Singapore	Breast	Adj.	CT (10-12 drugs)
Prospective Multicenter Study Evaluating Feasibility and Efficacy of Tumor Organoid-based Precision Medicine in Patients With Advanced Refractory Cancers (ORGANOTREAT)	NCT05267912	Jan-27	Paris, France	CRC, rare solid tumor	Pall.	CT
A Study on the Potential Benefit of Neoadjuvant Therapy for AGC Patients	NCT05442138	Sep-01	Henan, China	Gastric	NeoAdj.	UN
Functional Precision Oncology to Predict, Prevent, and Treat Early Metastatic Recurrence of TNBC (TOWARDS-II)	NCT05464082	Sep-27	Utah, USA	Breast	Pall. (2nd line)	CT
Precise Therapy for Refractory HER2 Positive Advanced Breast Cancer	NCT05429684	Feb-24	Shaanxi, China	Breast	Pall.	CT (10 drugs)
Using QPOP to Predict Treatment for Sarcomas and Melanomas (Q-SAM)	NCT04986748	Dec-28	Singapore	Sarcoma, melanoma	Pall.	CT (14 drugs)
Evaluation of ex Vivo Drug Combination Optimization Platform in Recurrent High Grade Astrocytic Glioma	NCT05532397	Dec-25	Singapore	Glioma	Pall.	CT

*Adj.* Adjuvant, *CT* Chemotherapy, *UN* Unknown, *NeoAdj.* Neaoadjuvant, *IT* Immunotherapy, *Pall.* Palliative, *RT* Radiotherapy, *TT* Targeted Therapy.

## Challenges and issues

**Fig. 4:**
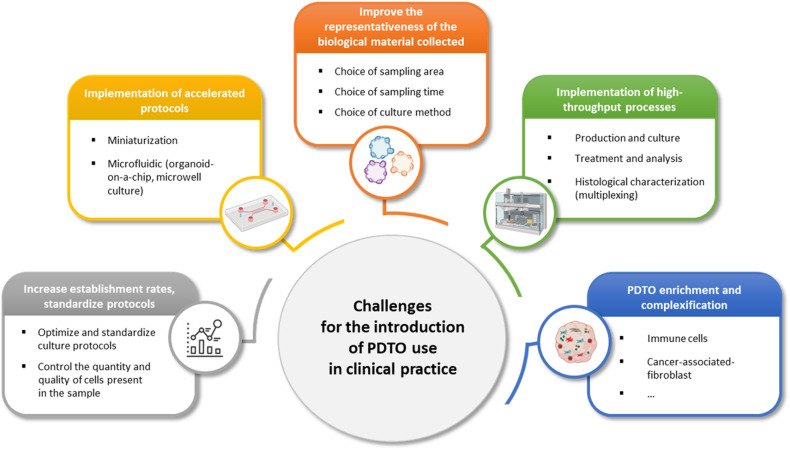
Future challenges for using PDTO in clinical practice (created with BioRender.com). Adapted from ^200^.

PDTO currently provides a wealth of information regarding its architectural organization, heterogeneity, molecular characteristics, and response to various treatments. The coherence of this response with clinical outcomes is gradually being confirmed by the literature. However, it is currently impossible to address all of the scientific and medical questions that have been raised by the community, due to a number of limitations of these models and/or their current culture conditions. In addition to the complexity of experimental protocols (which are difficult to routinely set up in a conventional biology laboratory) and the relative complexity of sampling circuits and associated regulatory and ethical aspects, serious limitations need to be addressed to ensure that reliable generated data are delivered to clinicians within a time frame that is compatible with clinical management. The challenges to be addressed include the representativeness of the sampled material considering the initial tumor and its polyclonal nature, the quantity of required material, the success and timing of establishment, the time needed to evaluate the response, the need to make the PDTO more complex or to at least culture them with stromal cells (such as fibroblasts and immune cells, among other cell types) to better predict the responses to some specific molecules, and the establishment of high-throughput culture processes capable of handling a large number of tumor samples (Fig. 4). Some of these points and potential solutions are discussed below.

### Ensuring the representativeness of the sample

One of the challenges in personalized medicine in oncology is ensuring that the utilized tumor material (in this case, PDTO) corresponds accurately to the tumor that will receive the treatment throughout the course of patient care. It has been demonstrated that liver PDTOs derived from different regions of the original tumor showed similar responses to different therapies. In contrast, ovarian PDTOs derived from distinct intraperitoneal nodules demonstrated treatment responses that could differ from each other^41^. These findings suggest the need to sample tumor material from multiple areas when tumors are disseminated and to generate as many clonal PDTOs as possible to ensure the representativeness of the obtained lineages. However, this would significantly complicate both the sampling and culture procedures. This issue needs to be evaluated in large-scale cohorts. Cancer cells can also evolve over time and under the influence of treatments to which they have been exposed. Previous studies have shown that the sensitivity of PDTOs derived from ovarian^41^, breast^99^ or pancreatic^100^ tumor samples at different stages of treatment is likely to change. It may be necessary to repeat tumor tissue sampling during disease progression, after treatment, in cases of relapse, or when metastases appear. Paradoxically, pancreatic PDTO derived from biopsies of recurrence or progression in patients who were already treated predicted the therapeutic response only 40% of the time, whereas PDTO derived from treatment-naive patients accurately predicted not only the response to the first line treatment (91%) but also to the second line (80%) of treatment^91^.

### Improving the success rate of establishment

The establishment rate of PDTO models varies considerably depending on the tumor location, ranging from less than 20% for prostate cancer^101^ to approximately 60% for ovarian cancer^22^ and up to over 90% for colon cancer^32^. The achievement of an establishment rate close to 100% will be necessary for the use of PDTOs to be feasible in a clinical context. Ooft et al.‘s study suggested that an insufficient establishment rate could be a major hindrance to the clinical use of PDTO. The proportion of tumor cells in the initial sample can influence the success rate of establishment^102^, thus potentially explaining the greater difficulty in obtaining a satisfactory establishment rate with small biopsies or after relapse. Furthermore, contamination by normal organoids from surrounding tumor tissues represents a real issue in some cases^103^. To prevent overgrowth of normal organoids, PDTOs can be selected by selective pressure based on their mutational pattern. For instance, the MDM2 antagonist Nutlin-3a can be used to select *TP53*-mutated PDTOs, and the withdrawal of EGF or Wnt3A can be used to select PDTOs harboring activating mutations in the EGFR and Wnt pathways, respectively^104^. PDTO can also be isolated from normal organoids via phenotype-based manual selection or via clonal expansion by using cell sorting. However, all of these approaches for selecting pure tumor organoids can lead to a loss of cellular heterogeneity compared to initial cultures^104^. Finally, for some types of tumors, such as sarcomas, the establishment of stable PDTO culture seems to be much more challenging^105^. Improvements in the establishment rate could involve enhancing the preparation and culture conditions (such as dissociation methods, culture substrates, and enriched or tailored media for the selection of tumor cells, among other conditions). The implementation of controlled and standardized methods is the first step in this process^14^. Fujii et al. achieved a 100% establishment rate by using eight different culture conditions, including various Wnt activators, p38 inhibitors, and oxygen concentrations. The varying requirements between tumors make it challenging to achieve a platform without overly costly or complex procedures^32^.

A platform based on more than 1000 PDTO models of different histological types was established to optimize culture conditions and analyze treatment responses^52^. The authors of that study showed that PDTOs can be established in both basic and enriched media (except for pancreatic tumors). However, subtle variations in the medium composition can sometimes have a significant impact on the establishment rate of tumor subtypes. It will likely be necessary to define the most suitable medium for each tumor type or subtype, such as by allowing for the best establishment rate and representativeness in a timely manner. Culture conditions could also be optimized by using finely controlled ECMs. Depending on the tumor type, the necessary biochemical and mechanical environments can vary considerably, thus suggesting the need for adaptation of the utilized matrices, as suggested by the optimization of a sliced tumor explant model^106^. The envisaged alternatives (whether they are natural, synthetic or a combination of both) show great potential but still require considerable developments to enable their widespread use and to completely replace current commercial matrices^107,108^.

### Making the predictive functional assay compatible with the clinical management timeline

One of the major limitations impeding the implementation of PTDO-guided therapeutic decision-making in routine clinical practice is how quickly the results are returned to the clinician for patient treatment. In the majority of cases, it is not compatible with the timeframe of clinical practice, and further technical challenges remain to be addressed to deliver a therapeutic option to physicians in a convenient time frame. This limitation can be overcome by increasing the elapsed time between sampling and patient treatment and/or making the predictive functional assay faster. For the first strategy, one option could involve generating PDTOs from biopsy at the time of diagnosis to inform the selection of adjuvant chemotherapy. However, this would require the processing and culture of some samples that will appear to be benign. Another option would be to use PDTO from treatment-naive patients to select the therapy that will be given after a recurrence or upon progression. Nonetheless, treatments exert selective pressures that drive tumor cell evolution and favor the appearance of resistant clones, thus leading to the development of recurrence with genetic profiles that are markedly different from those of the primary tumor. Therefore, further investigations are required to determine whether the response of PDTOs derived from samples that are collected prior to standard-of-care treatment could reflect the response to recurrence. The second strategy consists of decreasing the elapsed time between sampling and the results of the functional assay. The methods for treating PDTO and analyzing the response are crucial areas in which action can be taken to reduce response times. Many teams are specifically focusing on miniaturization and microfluidics processes, which would allow for the testing of a larger number of molecules on a smaller number of PDTOs, thus ultimately enabling more work on isolated PDTO (“single PDTO”) to accelerate evaluation of the response to treatment. This scenario is even more important because the amount of tumor sample is often drastically limited. In this context, the standardization of the methods also appears to be a major challenge because the variability of the response will increase if the number of PDTOs that are used for this evaluation is low. The influence of the number and size of PDTOs per condition on the response to treatment and the processes to be implemented are particularly important for controlling and standardizing these parameters. Further correlation studies will be required for these purposes.

For instance, by developing a microwell system allowing for the analysis of approximately one hundred PDTOs (a quantity obtainable in the first passage), a team recently evaluated the response to treatments of pulmonary PDTO within one week^109^. A microfluidic system leading to the formation of droplets of ECM around cells has also been proposed. PDTO was generated, and a response to functional testing was obtained in under 14 days, with the first correlations with clinical responses in patients being observed^110^. Another team conducted high-throughput screening one week after seeding ovarian tumor cells in an ECM matrix ring system rather than a droplet system^111^. Microfluidic devices based on the use of micrometer-sized channels also enable the dynamic control of nutrient, oxygen, and waste flows, thus consistently producing high-quality PDTOs^112^. The use of methods of treatment response analysis through imaging to accelerate information processing has also been proposed^113^. When combined with artificial intelligence, such methods have the potential to allow for rapid and cost-effective evaluation of treatment responses, thus further reducing the required response time. The development of new equipment automating the culture or treatment of PDTO, whole organoid sorting, and high-throughput microfluidic culture, among other methods, is also a subject of ongoing research, with biologists and physicists working together on these issues. Various automated techniques have been implemented by different platforms (such as the EIPM core facility in New York, https://eipm.weill.cornell.edu/research/organoids/; ORGAPRED core facility, www.orgapred.com and the laboratory of Stem Cell Bioengineering^114^). The aims of these initiatives are to work faster and with a smaller quantity of PDTOs so that results can be rapidly obtained (which is crucial for clinical use) and to allow for a greater number of tests to be conducted on the available PDTOs, such as by evaluating a larger number of molecules.

### Complexification of models

The interaction of cancer cells with cells in the tumor microenvironment, such as cancer-associated fibroblasts (CAFs), endothelial cells and immune cells, can influence treatment responses^115^ and/or constitute a therapeutic target. Anti-angiogenic strategies and immunotherapies are among the therapies for which it is still difficult to use PDTOs for predictive purposes. Various developments are currently underway to complexify PDTO models by coculturing them with cells from the tumor microenvironment, either directly or by using microfluidic devices enabling compartmentalized coculture of different cell types, as well as by using “vascularization”, or coculture of various types of normal cells and PDTOs (referred to as “organoids-on-chip, tumors-on-chip, organs-on-chip” approaches)^116,117^. These developments will accelerate preclinical evaluation and pharmacological research, in particular.

#### Immune cells

**Fig. 5:**
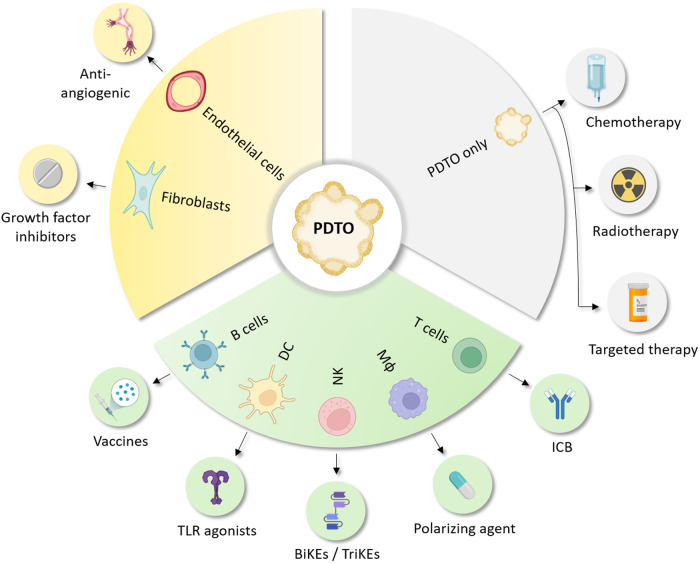
Coculture of PDTOs with autologous stromal and immune cells broadens therapies that could be tested. DC dendritic cell, NK natural killer cell, Mɸ macrophage, TLR toll-like receptor, BiKEs bispecific killer cell engagers, TriKEs trispecific killer cell engagers, ICB immune checkpoint blockade (created with BioRender.com).

As described above, one of the challenges in PDTO development is to “complexify” the culture with nontumoral cells to widen the spectrum of therapies with associated predictive assays (Fig. 5). Thus, the coculture of PDTOs with autologous immune cells is a very active field of research that aims to develop relevant models to evaluate and predict the responses to immunotherapies. A major issue in developing such a model involves the source of immune cells, as this affects their phenotype, including their maturation status, metabolism, cycling activity, and migration capacity. The use of immune cells infiltrating the tumor of origin seems to be the best strategy, as was proposed in a model of tumor slices cultured in ALI in which the immune diversity of the tumor microenvironment (TME) is maintained^36^. In addition to representing TME diversity, these types of models are sensitive to immune checkpoint blockade (ICB) and display morphologic changes and increased cell death after treatment^36,118–120^. PDTO using “native” immune cells can also be obtained from dissociated tumor samples cultured in Matrigel domes^121^ or acoustically assembled spheroids^122^ and are more easily cryopreserved. Nevertheless, due to the lack of immune-specific factors, immune cells infiltrating this type of PDTO exhibit a progressive decrease in viability and almost disappear after one month of culture^36^. Consequently, functional assays must be quickly performed, and the reuse of these models for further tests can be complicated. An alternative is to expand tumor-infiltrating lymphocytes (TILs), as suggested by the study by Knochelmann et al., who managed to isolate and expand TILs from murine and human solid tumors by using interleukin-2 (IL-2)^123^. This type of strategy has been successfully used with organoids and PDTOs derived from intestinal and colorectal tissue in which organoid infiltration and killing by intraepithelial T cells were observed^124–127^. However, the number of expandable TILs is highly dependent on the amount of tissue sample that is available, which may explain why this type of protocol is mainly used in the intestinal tract wherein the amount of resected tissue is relatively abundant. Thus, the use of peripheral immune cells may be needed for PDTOs derived from small pieces of resected tissue or biopsies. For example, Votanopoulos et al. used immune cells isolated from lymph nodes to activate patient-matched T cells to kill PDTOs derived from melanoma^128^, appendiceal cancer^129^ and Merkel cell carcinoma^130^. However, access to this type of surgical sample may not always be easy, which could prevent the use of immune-enriched PDTO on a larger clinical scale. Another option would be to use immune cells isolated from peripheral blood mononuclear cells (PBMCs), which may be easier to harvest. This method provided interesting results in PDTO derived from pancreatic ductal adenocarcinoma^131,132^ as well as colorectal and lung cancer^133^. Furthermore, these immune-enriched PDTOs using PBMCs may be more suitable for clinical purposes, as they were also used in an exploratory study (NCT03026140) assessing the response to neoadjuvant immunotherapy in colorectal cancers^134^. In this study, the authors managed to establish 12 PDTO-PBMC cocultures derived from patients and showed that T-cell reactivity against matched PDTO was more often observed in patients responding to treatment (3/6, 50%) than in nonresponders (0/6, 0%). Nevertheless, the use of such a model can induce a nonnegligible bias, as most of the T cells that are present in PBMCs will not display antigen specificity against PDTO. A first attempt to address this challenge was the study by Dijkstra et al., who published a detailed protocol in which repeated cycles of coculture of PBMCs and PDTOs were used to induce the emergence of PDTO-specific T cells^133–135^. Another challenge lies in the exhaustion status of the cells. Indeed, antigen persistence induces several alterations in T cells, such as immune checkpoint expression and epigenetic modifications, which have recently been reported to increase progressively from the periphery to the tumor bed^136^. Thus, preactivation protocols will have to mimic this exhaustion process as much as possible to increase the relevance of the model. Finally, until recently, immune-enriched PDTOs have mainly focused on T cells for the evaluation of immune checkpoint blockade (ICB)-based immunotherapies in clinical practice (Fig. 5). Complexification with other immune cells, such as macrophages, natural killer (NK) cells, dendritic cells (DCs) and B cells, may allow for other immunotherapies, such as macrophage polarizing agents, bispecific and trispecific killer engagers (BiKEs and TriKEs), Toll-like receptor agonists and cancer vaccines, to be tested (Fig. 5). Therefore, coculture of PDTOs and immune cells faces a number of challenges, which need to be addressed before an off-the-shelf model becomes available for translational and preclinical research.

#### Cancer-associated fibroblasts

CAFs play a significant role in tumor development and aggressiveness, which are primarily accomplished through the secretion of paracrine factors or ECM remodeling, thus providing both biochemical and mechanical support for tumor growth. However, long-term PDTO culture often leads to the gradual loss of multicellular components of the tumor microenvironment, thus limiting model accuracy^137^. Therefore, the development of PDTOs that can mimic in vivo cancer cell and stromal fibroblast interactions is crucial. These advances in the development of 3D coculture models of multicellular PTDO offer a deeper understanding of the cellular and molecular cues derived from both the cellular and acellular interactions provided by CAFs and their surrounding ECM. Organoids cultured in the presence of CAFs are useful for assessing complex diseases such as cancer. They can also be used to assess preclinical anticancer drugs prior to clinical trials. Recently, a 3D coculture of CAFs and oral cancer organoids was established. CAFs enhanced the organoid-forming ability of CD44+ oral cancer stem cells^138^. These cultures not only allowed us to evaluate the tumor-promoting effects of CAFs but also revealed the role of the NOTCH signaling pathway in the activation of CAFs^139^. A previous study demonstrated the relevance of incorporating CAFs in pancreatic PDTOs for the functional analysis of CAF activation. The authors identified the genesis of two CAF subpopulations, depending on the spatial localization within the pancreatic PTDO with different protein expression profiles^140^. CAFs may also exhibit antitumorigenic properties, as shown in lung squamous carcinoma PDTOs, in which epithelial overexpression of SOX2 is sufficient to mediate the transition from hyperplasia to dysplasia. Surprisingly, CAFs suppress the activity of high SOX2 levels, restore hyperplasia and enhance the formation of acinar-like structures, thus demonstrating that stromal factors can overcome cell-intrinsic oncogenic changes in determining the disease phenotype^141^. The addition of CAFs to liver PDTOs promoted tumor growth and resistance to conventional chemotherapeutic agents that are used in clinical practice. That study provided evidence for the potential clinical importance of CAFs in liver cancer^142^.

#### Vascularization

**Fig. 6:**
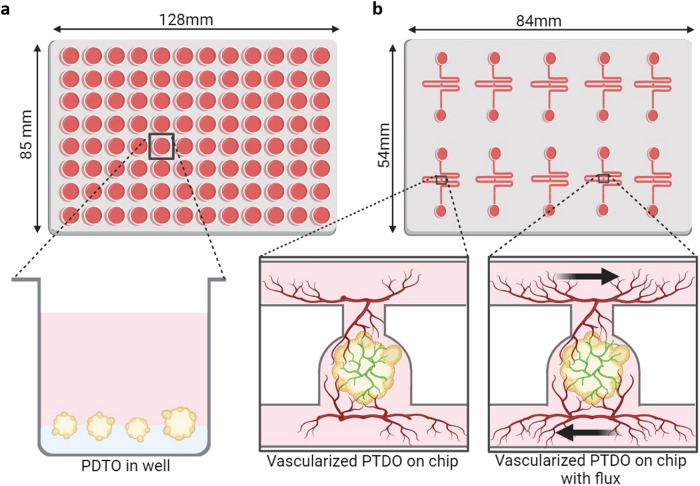
Vascularization and microfluidic applications for PDTO. **a** Representation of a classical 96-well plate with PDTOs grown in a Matrigel bed. **b** Representation of a serpentine microfluidic chip with a single PDTO vascularized with endothelial cells and an endothelial network in the gel, thus allowing for perfusion of the medium and/or drugs in the PDTO. The addition of flow improved vascular network formation (created with BioRender.com).

Coculturing PDTOs with endothelial cells allows for the analysis of the effectiveness of antiangiogenic molecules such as bevacizumab and sorafenib^143^ under conditions that are more similar to the physiology of the tumor. Indeed, as some tumors are particularly vascularized, whereas others are much less so (such as pancreatic cancer), it seems essential to consider this dimension when testing the potential efficacy of a treatment, particularly when evaluating intratumoral angiogenesis, the effect of cancer cells and the cancer microenvironment on tumor vascularization, endothelial network architecture and maturation dynamics and functionality. Microfluidics can significantly contribute to this domain, thus enabling work to be conducted under flow conditions that mimic the physiology of the tumor. This technology has been applied to several types of 3D culture, human induced pluripotent stem cells^144^, tumor spheroids^145^ and organoids^146^. The examination of the efficacy of a drug or immune cells on a vascularized PDTO under flow could better mimic the tumor environment, thereby improving the predictive capabilities of these models (Fig. 6).

These coculture models are particularly useful for some studies but necessitate modifications in the preparation of biological collections during PDTO preparation. It becomes important to preserve stromal cells from tumor dissociation as much as possible and to harvest autologous immune cells at the time of tumor sampling for subsequent coculture with autologous PDTOs. Although this procedure involves specific logistics and compliance with the rules and requirements for the use of human samples, it provides significant possibilities for applications, especially in clinical settings. Ongoing developments based on the use of coculture devices (such as organoids-on-chip) could allow for the evaluation of the response of PDTOs to a wide range of treatments, including those targeting the tumor microenvironment.

### Organoids-on-chip

The integration of PDTOs into microfluidic systems has subsequently emerged as being a powerful tool in cancer research. In addition to the advantages of PDTOs (as discussed above), microfluidics also have additional benefits for tumor organoids-on-chips, including precise control of nutrient and oxygen gradients, fluid flow, spatial organization, and the incorporation of components of the microenvironment, among other benefits, as well as the ability to create microphysiological systems (MPSs) that more closely resemble human physiology.

To date, numerous researchers have developed their own organoids-on-chip systems^145,147–149^, whereas others have used commercially available chips^150,151^. The overall design typically includes one channel with organoids embedded in a hydrogel and one or more channels of culture media, thus providing lateral flow by using a peristaltic pump or pressure controllers. Although these models typically overcome the issue of media renewal that comes with classical static cultures, most designs do not address issues such as normalization of organoid size, number and localization. To overcome these challenges, several teams have developed trapping methods by pipetting a single organoid into a central chamber^152^, with acoustofluidics^153^ or the hydrodynamic trapping of organoids^154^. Trapping methods allow for the standardization of the localization of the organoid, thus making image acquisition and downstream analysis more reproducible. In addition, the ability to trap a single organoid makes it possible to study tumor heterogeneity and clonal evolution, which is difficult to address with typical organoid culture methods.

In terms of cancer research applications, organoids-on-chip can help with various issues that the field is experiencing. First, high-throughput screening of anticancer drugs can be facilitated by simultaneously testing multiple compounds, along with different combinations of treatments or even drug regimens^155,156^. These platforms also enable the development of personalized cancer therapies by culturing PDTOs and testing drug responses ex vivo. Due to the limited quantity of biological samples that are needed, the delay between the procurement of biopsies and the response to drug treatments from mature organoids can decrease, which is one of the greatest challenges in the field of personalized medicine. Additionally, organoids-on-chip better emulate the tissue microenvironment than do organoids.

The addition of flow itself is an important microenvironmental cue that benefits organoid growth over culture in well plates. For example, the size and efficiency of PDTO formation increased with the addition of flow in a colorectal cancer context^157^. Another team observed improvements in ovarian cancer organoid size and changes in response to drugs^158^. Moreover, the integration of vascular or endothelial networks inside of organoids-on-chip models represents a major advantage over classic PDTO culture and enables researchers to specifically examine intratumoral angiogenesis, the effect of cancer cells and the cancer microenvironment on tumor vascularization. The endothelial network inside of the organoids-on-chip system usually surrounds the organoid or PDTO^159^ and can anastomose with the 3D structure, thus providing successful intravascular perfusion (Fig. 6) and, as a consequence, better maturation of the organoid^146^. Moreover, the endothelial network is more developed when there is media flow and can be perfused and transport small molecules^160^, blood cells^161^ or PBMCs^162^. The circulation of cells and small molecules provides the opportunity to study not only cancer metastasis phenomena but also tumoral inflammation or immunotherapies. Finally, with appropriate platforms, on-chip organoids can be coupled with sensors to obtain more quantitative data and perform real-time kinetic studies on the response of PDTOs to treatments. For instance, such captors could measure oxygen levels^163^ or metabolites such as glucose or lactate^164^ to more accurately evaluate PDTO metabolism.

By combining the strengths of PDTO culture with microfluidic engineering, this innovative platform allows for the improvement of tissue differentiation and integration of microenvironmental cues into PDTOs. Overall, organoids-on- chip systems exhibit great potential for accelerating drug discovery, understanding disease mechanisms, and ultimately improving patient outcomes in oncology.

## Conclusion

Organoids and PDTOs represent groundbreaking developments for both researchers and clinicians in various fields, including developmental and cancer biology, regenerative medicine, toxicology, drug development, and precision oncology. Their vast potential is waiting to be fully exploited, although numerous challenges remain for the scientific and medical communities. These challenges include an understanding of how to successfully obtain and maintain PDTOs, accelerating establishment processes and predictive testing, enhancing model complexity, and improving physiological representation via enrichment or integration into coculture devices. High-throughput culture and analysis processes, protocol standardization, and determination of the optimal sampling for obtaining reliable responses are challenges that are being actively addressed by the scientific community. With the integration of these models into routine clinical use, precision oncology can enter into a new era in the coming decade. This represents the essence of research that is being conducted worldwide and ongoing clinical trials aimed at validating the potential of this approach. The future holds considerable promise for leveraging these models to advance personalized cancer treatment and transform patient care.
